# Fellowships, Grants, & Awards

**Published:** 2005-06

## Japan Society for the Promotion of Science: Opportunities for U.S. Scientists in Japan

The Japan Society for the Promotion of Science (JSPS) conducts fellowship programs for foreign researchers to promote international cooperation in and mutual understanding through scientific research in Japan. These programs provide opportunities for U.S. researchers to conduct cooperative research under their host researchers. Research applications are accepted at the John E. Fogarty International Center of the National Institutes of Health, which acts as a nominating authority for JSPS programs. As the eligibility requirements and application procedures are different for each fellowship, the following information describing eligibility and application procedures should be reviewed before submitting an application.

### JSPS Invitation Fellowships.

The JSPS conducts short- and long-term programs under the Invitation Fellowship Program, funded by a subsidy from the Japanese government. The fellowship allows scientists employed at designated Japanese research institutions and laboratories to invite fellow researchers from the United States to Japan to participate in cooperative activities. These visits presuppose the existence of contacts between scientists in Japan and other countries, a condition considered favorable to the promotion of future scientific cooperation and exchange. Application deadline: 31 October 2005.

### JSPS Postdoctoral Fellowships.

The JSPS conducts short- and long-term Postdoctoral Fellowships for Foreign Researchers. These fellowships were established to assist promising and highly qualified young researchers wishing to conduct research in Japan. The program is aimed at providing opportunities for such researchers to, under the guidance of their hosts, conduct cooperative research with leading research groups in Japanese universities and other institutions, thereby permitting them to advance their own research while stimulating Japanese academic circles—particularly young Japanese researchers—through close collaboration in scientific activities. The program’s intention is also for such collaboration to advance scientific research in the counterpart countries. Postdoctoral Fellowship (short-term) application deadline: 31 October 2005. Postdoctoral Fellowship (long-term) application deadline: 30 June 2005.

Contact: Maria “Mili” Ferreira, Division of International Training and Research, Fogarty International Center, NIH, 31 Center Dr, Bldg 31, Rm B2C39, MSC 2220, Bethesda, MD 20892-2220 USA, 301-594-9778, fax: 301-402-0779, e-mail: ferreima@mail.nih.gov.

## Arsenic Research: The Grainger Challenge Prize for Sustainability

The National Academy of Engineering (NAE), supported by The Grainger Foundation, has established the Grainger Challenge Prize for Sustainability. The primary purpose of this “inducement prize” is to accelerate the development and dissemination of technologies to enhance social and environmental sustainability for the benefit of current and future generations. A complementary goal of the prize competition is to increase awareness among the U.S. engineering community of the importance of designing and engineering for sustainability, particularly in an international context, and to encourage and showcase efforts by U.S. engineers to bring sustainable technologies to the marketplace and promote green design philosophies. The recipient(s) of the prize will be awarded US$1 million.

The specific goal of this competition, which may be followed by future prize competitions in like amounts for comparable goals, will be the development of a community- or household-scale water treatment system to remove arsenic from the contaminated groundwater found in many developing countries. The system must have a low life-cycle cost; must be technically robust, reliable, maintainable, socially acceptable, and affordable; must be manufactured and serviced in a developing country; and must not degrade other water quality characteristics.

Arsenic contamination has affected millions of people, primarily in rural Bangladesh, as well as in eastern India, Nepal, and several other countries. In Bangladesh, the arsenic is an unintended consequence of an aggressive international program to control the spread of cholera spread through surface waters by drilling thousands of tubewells. Unfortunately, the tubewells tapped into aquifers containing hundreds of parts per billion (ppb) of naturally occurring arsenic, usually within 100 meters of the surface.

Efforts to solve this problem have been under way for a decade, but no single solution has been implemented on a large scale. Field and laboratory tests have been conducted on technologies to determine if they are affordable, robust, and meet World Health Organization (WHO) water quality standards for a treatment system that can be used either in individual homes or at the community level. The intent of the NAE/Grainger Foundation competition is to encourage the U.S. engineering community to become engaged in finding a solution to this specific challenge.

### Technical Performance.

The successful technology should address the potable water needs of a rural community of approximately 1,000 residents (roughly 200–300 families). Daily per capita potable water demand is assumed to be 7.5 liters as recommended by the WHO. Such a community might be served by a community-level system, with water piped to convenient distribution points or into homes, or by hand-operated tubewell pumps spread throughout the community. Arsenic can be removed and controlled either at a central community plant or at the household level.

In typical villages, women and children usually fetch water. Therefore a treatment process will depend largely on women’s and children’s ability to use the system easily and conveniently. However, community-level systems could be run by a trained operator. Contestants may submit proposals for either type of system.

As the reference standard of contamination, specially formulated test water will be used with an arsenic concentration of 300 ppb. This level of contamination is considered to be in the midrange in severely affected areas. The system must reduce this concentration to 10 ppb—presently considered the most acceptable standard by the U.S. Environmental Protection Agency and the WHO—without causing deterioration in other water quality characteristics such as taste and odor, or resulting in an increase in fecal coliforms or other contaminants. The exact formulation of the test water and the complete testing protocol will be made available by the NAE. Contestants may assume that electricity is available for community-level systems because rural areas in many developing countries do have electric power. However, a point-of-use system that does not require electricity will also be considered. If electricity is required, the full life-cycle cost of providing it in all locations must be factored into the economic feasibility estimate.

### Economic Performance/Scalability.

On a per-person basis, the winner will maximize the volume per unit time while minimizing the cost and arsenic content. Because 10 ppb is the WHO standard, no extra credit will be given for attaining lower concentrations. Obviously, this metric allows for some tradeoffs. However, the other constraints described in the Technical Performance section above must not be compromised.

All system components should be capable of being manufactured and serviced locally at the village level. Contestants must develop a business plan to demonstrate how this might be done in a specific rural environment and must demonstrate the potential for scale-up to thousands of units. Contestant must also have a plan for disseminating the technology through a nongovernmental organization, the private market, or government, and for covering all costs. All technology developed by the contestants will remain their property. The NAE will hold all information conveyed to it by the contestants in confidence, except that the winning contestant must agree to disclose the winning technology at the time of announcement of the award in February 2007. It is the sole responsibility of all contestants to apply for such patent protection as they deem necessary.

### Social Acceptability.

Social acceptability must be demonstrated either through carefully documented field experience and/or by drawing upon existing research in social/behavioral science relevant to the challenge. Each contestant must submit a credible field monitoring plan describing how well the system is expected to work, and over what period of time and at what level of maintenance. The plan should also pinpoint expected operational problems and explain how they might be handled.

### Judging.

Entries will be judged by a panel of NAE members appointed by the NAE Council. Panel members will be prominent members of the engineering, scientific, industrial, government, educational, and environmental communities. The panel will base its selection of a winner on the criteria outlined in the three requirements sections above with the assistance of specialists familiar with the arsenic problem.

Under the conditions of the NAE/Grainger Foundation Prize Agreement, the Grainger Prize is available only to a U.S. citizen or team of U.S. citizens, who must be living at the time of notification of selection as prize recipients in the United States. All team members must agree and stipulate in writing in advance how they wish the prize money to be distributed. Such stipulation, once made, shall be irrevocable and unchangeable. The NAE accepts no responsibility for adjudicating disputes of any kind among team members. All expenses incurred by the contestants in pursuit of the prize are their responsibility. For the most up-to-date announcement, see http://www.nae.edu/nae/grainger.nsf/weblinks/NAEW-68HUZT?OpenDocument.

Contact: Jack Fritz, NAE, 500 Fifth Street NW, Washington DC 20001 USA, 202-334-2491, e-mail: jfritz@nae.edu.

## Global Research Initiative Program, Social Science

As part of its global health initiative, the John E. Fogarty International Center (FIC) of the National Institutes of Health (NIH), in partnership with the National Eye Institute (NEI), the National Heart, Lung, and Blood Institute (NHLBI), the National Institute on Drug Abuse (NIDA), the NIEHS, the National Institute of General Medical Sciences (NIGMS), the Office of Behavioral and Social Sciences Research (OBSSR), the Office of Dietary Supplements (ODS), and the Office of Research on Women’s Health (ORWH), invites applications from current and former NIH-supported foreign research trainees to compete for funds that will support their research efforts upon return to their home countries.

As junior scientists complete training programs in the United States, many find it difficult to secure the support needed to continue their research projects and careers in their home countries. This Global Research Initiative Program (GRIP) provides the opportunity for junior foreign scientists to compete for such funds through a peer-reviewed process. This is a critical adjunct in the continuation of promising independent research careers that will be of benefit to the investigators’ home countries and the world at large. Women and underrepresented minority scientists in their countries are especially encouraged to apply for these reentry grants. Project proposals should be geared toward the research interests of the applicant and focus on high-priority health and health care problems in the investigator’s home country that also carry global importance, and are of interest to the collaborating institutes, centers, and offices.

In order to be eligible, foreign scientists must meet at least one of the following criteria: 1) at least two years of research training experience under an FIC-supported training grant; 2) one year of such training experience coupled with one year of significant, well-documented mentored research experience; 3) one year of the NIDA INVEST Fellowship plus at least one additional year of mentored research (http://www.drugabuse.gov/International/HHHRF.html); 4) at least two years of research training experience through the NIH intramural Visiting Fellows Program; 5) one year of training through an f5 international fellowship program and one subsequent year of mentored research; 6) be a recipient of a Long-Term Fellowship award through the Human Frontier Science Program, who comes from a low- or middle-income country, and who has spent at least two years in research training; or 7) at least one year of training in the United States and one additional year of significantly mentored research, in the United States or abroad, leading to a completed master’s degree or doctoral degree, at least partially funded through an FIC research training program, with preapproval by the program director.

It is expected that research topics will be diverse. Please refer to the full program announcement at http://grants.nih.gov/grants/guide/pa-files/PAR-05-082.html for more information on specific research topics of interest. All research must be performed in accordance with NIH and U.S. government regulations regarding the responsible conduct of research. This program precludes the support of research involving enrollment in pilot studies for clinical trials or the actual support of clinical trials since the resources and infrastructure to support and oversee such trials generally exceed the resources available under this award mechanism.

Evaluation of the program will occur on an ongoing basis. Because this is a program to move research trainees to the status of independent investigator, there are several outcomes to be measured: 1) development of laboratory capabilities or research projects; 2) training of other potential researchers; 3) publications in local as well as international peer-reviewed journals; 4) participation in workshops, seminars, and international conferences; 5) collaborations with past mentors, as well as with other researchers; and 6) attraction of funding from other sources.

This funding opportunity will use the R01 award mechanism. An applicant can request up to two modules of $25,000 each or total direct costs of $50,000 per year, plus facilities and administrative costs to a maximum of 8% for a foreign institution. Applications may have a project period of no less than three years and no more than five years. Because an investigator can receive a maximum of five years of support under the GRIP program, and this specific GRIP award is not renewable, any future application will be considered to be an unsolicited competing application based on this project and will compete with all investigator-initiated applications submitted to NIH through the Center for Scientific Review.

Applications must be prepared using the PHS 398 research grant application instructions and forms. Applications must have a Dun and Bradstreet (D&B) Data Universal Numbering System number as the universal identifier when applying for federal grants or cooperative agreements. The D&B number can be obtained by calling -866-705-5711 or online at http://www.dnb.com/us/. For further assistance contact GrantsInfo by calling 301-435-0714 (telecommunications for the hearing impaired: TTY 301-451-0088) or by e-mail: GrantsInfo@nih.gov.

The letters of intent receipt dates for this PAR are 22 August 2005; 21 August 2006, and 21 August 2007, with the application receipt dates 21 September 2005; 21 September 2006; and 21 September 2007. The earliest anticipated start date for these awards is July of the year following the receipt date.

Contact: Aron Primack, Division of International Training and Research, FIC, Bldg 31, Rm B2C39, 31 Center Dr, MSC 2220, Bethesda, MD 20892-2220 USA, 301-496-4596, fax: 301-402-0779, e-mail: primacka@mail.nih.gov; Chyren Hunter, Retinal Neurosciences and Oculomotor Systems Program, Division of Extramural Research, NEI, 5635 Fishers Ln, MSC 9300, Ste 1300, Bethesda, MD 20892-9300 USA, 301-451-2020, fax: 301-402-0528, e-mail: clh@nei.nih.gov; Ruth Johnsson Hegyeli, Office of the Director, NHLBI, NIH, 31 Center Dr, Rm 4A07, Bethesda, MD 20892-2490 USA, 301-496-5375, fax: 301-496-2734, e-mail: hegyelir@nih.gov; Steven Gust, International Programs, NIDA, 6001 Executive Blvd, Rm 5-274, Bethesda, MD 20892-9581 USA, 301-443-6480, fax: 301-443-9127, e-mail: ipdirector@nida.nih.gov; Dennis Lang, Division of Extramural Research and Training, NIEHS, PO Box 12233, MD EC-20, Research Triangle Park, NC 27709 USA, 919-541-7729, fax: 919-541-2583, e-mail: lang4@mail.nih.gov; Ann A. Hagan, NIGMS, 45 Center Dr, Rm 2AN24H, Bethesda, MD 20892-6200 USA, 301-594-4499, fax: 301-480-1852, e-mail: hagana@nigms.nih.gov; Virginia Cain, Office of the Director, OBSSR, Bldg 1, Rm 256, 1 Center Dr, Bethesda, MD 20892 USA, 301-402-1146, fax: 301-402-1150, e-mail: virginia_cain@nih.gov; Mary Frances Picciano, ODS, 6100 Executive Blvd, Ste 3B01, Bethesda, MD 20892-7517 USA, 301-435-3608, fax: 301-480-1845, e-mail: piccianm@od.nih.gov; Lisa Begg, Office of the Director, ORWH, 1 Center Dr, Rm 201, Bethesda, MD 20892 USA, 301-402-1770, fax: 301-402-1798, e-mail: beggl@mail.nih.gov. Reference: PAR-05-082

